# Pharmacological evaluation and phytochemical profiling of butanol extract of *L. edodes* with in- silico virtual screening

**DOI:** 10.1038/s41598-024-56421-7

**Published:** 2024-03-08

**Authors:** Umer Ejaz, Muhammad Afzal, Muhammad Naveed, Zeemal Seemab Amin, Asia Atta, Tariq Aziz, Gul Kainat, Noshaba Mehmood, Metab Alharbi, Abdullah F. Alasmari

**Affiliations:** 1 Department of Biochemistry, Faculty of Science and Technology, University of Central Punjab, Lahore, 54590 Pakistan; 2 School of Biochemistry, Faculty of Applied Sciences, Minhaj University Lahore, Lahore, 54590 Pakistan; 3 Department of Biotechnology, University of Central Punjab, Lahore, 54590 Pakistan; 4 Department of Biochemistry, Nur international university, Lahore, 54590 Pakistan; 5 Laboratory of Animal Health Food Hygiene and Quality, University of Ioannina, 47132 Arta, Greece; 6 Department of Microbiology, University of Central Punjab, Lahore, 54590 Pakistan; 7 Department of Pharmacology and Toxicology, College of Pharmacy, King Saud University, P.O. Box 2455, 11451 Riyadh, Saudi Arabia

**Keywords:** Invitro, Insilico analysis, *L.edodes*, Phytochemicals, Ruminated arthritis, Biochemistry, Biological techniques, Biotechnology, Chemical biology, Computational biology and bioinformatics, Drug discovery, Microbiology, Molecular biology, Structural biology

## Abstract

*L. edodes* (*L. edodes*) is the most consumed mushroom in the world and has been well known for its therapeutic potential as an edible and medicinal candidate, it contains dietary fibers, vitamins, proteins, minerals, and carbohydrates. In the current study butanolic extract of mushroom was used to form semisolid butanol extract. The current study aimed to explore biometabolites that might have biological activities in *n*-butanol extract of *L. edodes* using FT-IR and GC–MS and LC–MS. The synergistic properties of bioactive compounds were futher assessed by performing different biological assays such as antioxidant, anti-inflammatory and antidiabetic. FTIR spectra showed different functional groups including amide N–H group, Alkane (C-H stretching), and (C = C stretching) groups at different spectrum peaks in the range of 500 cm^−1^ to 5000 cm^−1^ respectively. GC–MS profiling of *n*-butanol extract depicted 34 potent biomolecules among those dimethyl; Morphine, 2TMS derivative; Benzoic acid, methyl ester 1-(2-methoxy-1-methylethoxy)-2-propanol were spotted at highest range. Results indicate *that L. edodes n-*butanol extract showed a maximum anti-inflammatory potential 91.4% at 300 mg/mL. Antioxidant activity was observed by measuring free radical scavenging activity which is 64.6% at optimized concentration along with good antidiabetic activity. In-silico study executed the biopotential of active ingredient morphine which proved the best docking score (− 7.0 kJ/mol) against aldose reductase. The in-silico drug design analysis was performed on biometabolites detected through GC–MS that might be a potential target for sulfatase-2 to treat ruminated arthritis. Morphine binds more strongly (− 7.9 kJ/mol) than other bioactive constituents indicated. QSAR and ADMET analysis shown that morphine is a good candidates against ruminated arthritis. The current study showed that *L. edodes* might be used as potent drug molecules to cure multiple ailments. As mushrooms have high bioactivity, they can be used against different diseases and to develop antibacterial drugs based on the current situation in the world in which drug resistance is going to increase due to misuse of antibiotics so new and noval biological active compounds are needed to overcome the situation.

## Introduction

A remarkable amount of interest has been attracted to wild mushrooms in recent years in medicine and food processing because of their outstanding nutritional and therapeutic qualities. Over 14,000 species of mushrooms are known worldwide, of which about 2000 are edible^1^. There are also over 200 varieties of mushrooms that are grown commercially for Ayurveda medicine synthesis and human consumption^2^. Mushrooms have been known as a good source of nutrients and might be used to promote promising effects in boosting immunity to improving health. Moreover, they contain high levels of dietary fibers, low in calories and carbohydrates, and high amount of protein containing all the essential amino acids, minerals, and vitamins, with low fats contents and cholesterol^3,4^. Cultivated medicinal mushrooms were used to treat minor illnesses and epidemic diseases, whereas edible mushrooms have a delightful taste, aroma, texture, and flavor^5^. Pharmaceutical benefits over the last decade were observed, according to some researchers, mushrooms contain compounds that indicate these compounds have anti-inflammatory, antioxidant, and antimicrobial properties. For example, some medicinal mushrooms have been found to have the ability to inhibit the growth of certain bacteria and reduce inflammation in the body and can be utilized to treat a variety of ailments^6^. Cancer is a complex disease that requires a multi-faceted approach to combat. Therefore, current drug discovery efforts are aimed at targeting multiple mechanisms involved in cancer growth, such as mutations and biochemical signals^7,8^.Secondary metabolites produced by mushrooms have therapeutic potential. In addition to their antioxidant, antimicrobial, anticancer, cholesterol-lowering, and immune-stimulating properties, mushrooms possess a wide range of health-promoting properties^8^. *L. edodes* was used because of its great nutritional and therapeutic properties described in previous literature^9^. *L. edodes* is used as a natural resource because it has many useful bioactive compounds to combat the inflammation, diabetes and free radicals detected through GC–MS and LC–MS. Previous literature demonstrated that secondary metabolites were found in mushrooms such as *Ganoderma lucidum*, Oyster, *Lentinua edodes* and Lingzhi mushrooms which are responsible for their therapeutic effects^10^.

The immune system responds to inflammation by responding to physical, chemical, and pathogenic factors. Usually, acute inflammation is a short-term process that resolves naturally. Inflammation may occasionally progress to chronicity^11^. Physiological factors such as aging and deficiency in antioxidants, vitamins, and anti-inflammatory elements (zinc, and selenium) may contribute to inflammation persistence. Besides neurodegenerative diseases and cancer, chronic inflammation can also lead to autoimmune diseases^12^. Reactive oxygen species (ROS) is a molecule that is highly reactive because of oxygen metabolism. ROS are produced as a metabolite during cell and tissue activity at normal physiological concentrations in case of infections and degenerative diseases, such as cardiovascular disease, aging, and neurodegenerative diseases like Alzheimer’s, mutations, and cancer. However, ROS can cause damage when they are present at high concentrations^13^. Diabetes mellitus is characterized by a high blood glucose level that damages the micro- and macrovascular systems in the body^14^. Secondary metabolites, including polysaccharides, steroids, terpenes, and peptides, are produced by both edible and medicinal mushrooms. Some of these metabolites are medicinally significant, such as antioxidants, anti-tumors, anti-diabetics, anti-cancers, anti-aging, and anti-obesity. Additionally, mushrooms’ secondary metabolites are prioritized over other natural compounds since they’re non-toxic and have minor or no side effects. Thus, various secondary metabolites are produced by mushrooms, which is advantageous in the preparation of new drugs^15^. A mushroom commonly referred to as shiitake is *L. edodes* (shiitake) which belongs to the family Omphalotaceae Basidiomycota and is widely cultivated and eaten in many Asian countries as well as in Europe*.* The species possesses medicinal properties, which are primarily used in traditional medicine but are also used in conventional oncology practices^16^.

For the characterization and recognition of chemicals or functional groups (chemical bonds) contained in an unknown blend of mushroom extract, FT-IR has shown to be a useful tool. A chemical “fingerprint” can be made from the FT-IR spectra of pure substances since they are typically so 22 distinctive. It is possible to determine the spectrum of most common plant and mushroom chemicals by linking the spectrum of an unknown compound to a library of known compounds. There are numerous techniques to prepare samples for FT-IR^17^. The amount of different metabolites can be performed with GC–MS. This includes a variety of volatiles that may all be directly measured, including ketones, aldehydes, alcohols, heterocyclic compounds and hydrocarbons with up to 12 carbons. There is currently a wide variety of instruments available with varying ionisation types and mass separations. They are quite inexpensive compared to other devices and come with a number of benefits, including great resilience, high sensitivity, and high precision of the observed labelling patterns. The GC intake receives the sample solution, which is typically between 0.1 and 1 L in volume. The carrier gas, often helium, vaporises the sample solution and directs it onto the chromatographic column. The capillary column should be wisely designated in order to get the maximum resolution possible in GC–MS analysis^18^. A significant advantage of LC/MS over GC/MS has been the absence of the need to derivatize the samples, and the combined technology is already seeing use in metabolic profiling when the right polarity and mobile phase compositions are used. Based on the biological activity of the extracts, LC MS (LTQ XL, Thermo Electron Corporation, USA) analysis was performed to identify bioactive compounds.A medicinal and edible mushroom, the *L. edodes* (shiitake) is widely cultivated throughout the world*.* The active compounds in the mushroom have been studied and found to have anti-inflammatory, anti-tumor, anti-viral, and anti-bacterial properties. In addition, shiitake mushrooms contain high levels of proteins, vitamins, and minerals, making them an excellent choice for those looking to supplement their diets with nutritious food*. L. edodes* is considered to be one of the most valuable medicinal mushrooms^19^. There has been extensive research into its biological activity including antitumor, immune modulation, antiviral, antibacterial, cholesterol regulation, anti-atherosclerosis, diabetes prevention, and antioxidant effects. Because of its wide range of beneficial effects, this mushroom has significant potential as a natural medicine^20^. The current study aims to evaluate FT-IR, GC–MS and LC–MS analysis of numerous phytochemical constituents present in the *n*-butanol extract of *L. edodes*, furthermore, In-vitro biological studies of the extracted mushroom were performed, including anti-inflammatory, antioxidant, and anti-diabetic assessments. The computational validation was also performed to identify the phytochemicals responsible for the therapeutic effects on ruminated arthritis (RA) and in vitro anti-diabetic activity.

## Material and methods

### Description of study area

The *L. edodes* confirmed strain M390 was grown in Mushroom Lab of University of Agriculture Faisalabad in Faisalabad, and it is the second largest district of province Punjab-Pakistan.

### Mushroom collection and preparation

*L. edodes* mushroom samples (500 g) were collected from University of Agriculture Faisalabad. To dry the mushroom, it was cleaned, cut into small pieces, and dried at 40 °C for 15 to 20 h. The powdered material was stored at room temperature in an airtight plastic bag in a desiccator to facilitate further analysis.

### Extraction method

To extract biometabolites from *L. edodes n*-butanol extraction was performed at 37 °C for 5 days with a ratio of mushroom powders to *n*-butanol of 1:3 in which 500 g sample soaked in 1500 mL of butanol. Using Whatman No. 1 filter paper, solids were filtered out. *n*-butanol was removed to yield the semi solid extract from the filtrate by rotary evaporator, and then 7 g of crude extract of *L. edodes* was obtained. At a concentration of 1 g/mL, the crude was dissolved in dimethyl sulfoxide (DMSO). For this experiment, *n*-butanol extract was kept at 20 °C in a glass bottle^21^.

### FT-IR spectroscopic analysis

Fourier transform infrared spectrophotometers (FT-IR) are highly effective tools for identifying chemical bonds in compounds. The Fourier transform infrared spectroscopy(FT-IR) study of *L. edodes n-*butanol extract was performed by using the Perkin Elmer spectrometer system to find out characteristic peaks and functional groups at a resolution of 4 cm^−1^ within peak range of 500–4500 cm^−1^^22^.

### Gas chromatography- mass spectrometry (GC–MS)

GC–MS analysis was carried out using a 7890A gas chromatograph (Agilent 19091-433HP, USA) and mass spectrophotometer, equipped with a HP-5 MS fused silica column (5% phenyl methyl siloxane, 30 × 250 μm, film thickness 0.25 μm), interfaced with 5675CInert MSD with Triple-Axis detector. With helium gas as the carrier gas, the column velocity was set to 1.0 mL/min. In addition to the GC–MS parameters, the ion source temperature is 250 °C, the interface temperature is 300 °C, the pressure is 16.2 psi, and the out-time is 1.8 mm. Special parameters include a split ratio of 1:50 with a 300 °C interface temperature, and another data point is the temperature of the interface. Within five minutes of being maintained at 36 ºC, the temperature of the column increased gradually from 36 to 150 ºC. A temperature increased up to 20 °C/minute for five minutes was used to reach 250 °C. The elution took 47.5 min to complete. By comparing each component’s average peak area with its total area, we calculated its percentage amount. Data was collected using The supplier used MS Solution software for controlling and monitoring the system^21^. These compounds can be identified using NIST MS 2.0 libraries.

### Column chromatography

Butanolic extract was further fractionated to purify biological active compounds by loading silica gel 400–600 µm pore size in a glass column by selecting different solvents to set polarities according to the nature of the fraction to collect the column fraction for further purification of secondary metabolites by passing extract through the column. Partially purified biometabolites were determined in the collected fraction by finding out their retention time, percentage area, molecular weight and monoisotpoic mass by performing LC-MS analysis.

### LC–MS analysis

LC–MS was employed for non-volatile and high molecular weight bioactive compounds in the *n*-butanol extract passed through the column. A triple quadrupole liquid chromatography-mass spectrometry system (Finnigan TSQ Quantum Ultra EMR, Thermo Scientific) was employed in the study^23^.

### Biological activities

#### In vitro* anti-inflammatory potential*

A slightly modified standard protocol was used to test in-vitro anti-inflammatory potential^24^. The protein denaturation inhibition method was used to study *L. edodes n*-butanol extract*.* To adjust pH from 8.53 to 6.74, Bovine serum albumin (BSA) (0.4%) was dissolved in tris buffer saline with glacial acetic acid^25^. The reaction mixture consisted of BSA (0.2 mL), 1X PBS (Phosphate-Buffered Saline) (2.8 mL) (pH: 6.4), and each mushroom extract (2 mL) in varying concentrations of 100,150,200,250 and 300 mg/mL. A similar volume of distilled water was used as a control. Incubation of the mixture at 37 °C for 15 min was followed by heating on a water bath for 5 min at 70 °C. Samples were cooled before being measured at 660 nm. A reference drug that was used in this study was diclofenac sodium (1 mg/mL). Using the following formula calculate the percentage of protein denaturation inhibition:

$$\% \,{\text{inhibition}} = \frac{{\left( {{\text{Absorbance}}\,{\text{of}}\,{\text{sample}} - {\text{Absorbance}}\,{\text{of}}\,{\text{negative}}\,{\text{control}}} \right)}}{{{\text{Absorbance}}\,{\text{of}}\,{\text{positive}}\,{\text{control}}}} \times 100$$

#### Antioxidant activity (DPPH)

A determination of the radical scavenging activity of DPPH (2,2-diphenyl-1-picrylhydrazyl) was performed at different concentrations (100,150,200,250,300 mg/mL) of *L. edodes n*-butanol extract^26^. The reaction mixture contained 0.5 mL of extract, 3 mL of butanol, and 0.3 mL of 0.5 mM 2,2-diphenyl-1-picrylhydrazyl (DPPH) radical solution in methanol. An absorbance measurement at 517 nm was performed after incubation for 45 min. The following equation was used to calculate antioxidant activity.

$$\% \,{\text{inhibition}} = \frac{{\left( {{\text{Absorbance}}\,{\text{of}}\,{\text{sample}} - {\text{Absorbance}}\,{\text{of}}\,{\text{negative}}\,{\text{control}}} \right)}}{{{\text{Absorbance}}\,{\text{of}}\,{\text{positive}}\,{\text{contol}}}} \times 100$$

#### Anti-diabetic activity

The protocol given by Dessalegn et al., 2019 indicated to possess antidiabetic activity by DNSA (3,5-dinitrosalicylic acid**)** assay at different concentrations (100,150,200,250,300 mg/mL) of *L. edodes n*-butanol extract. 200 µL extract of *n*-butanol is mixed with 200 uL α-Amylase solution. A reaction mixture was incubated at 25 °C for 10 min. After incubation, 200 µL of 1% starch solution was added and the mixture was incubated for another 10 min. Following this, 400 µL of DNSA solution is added and the absorbance of the mixture is measured by ELISA at 630 nm. Metformin was used as the standard and distilled water as a negative control^27^.

$$\% \,{\text{inhibition}} = \frac{{\left( {{\text{Absorbance}}\,{\text{of}}\,{\text{sample}} - {\text{Absorbance}}\,{\text{of}}\,{\text{negative}}\,{\text{control}}} \right)}}{{{\text{Absorbance}}\,{\text{of}}\,{\text{positive}}\,{\text{contol}}}} \times 100$$

### Molecular Docking

#### Preparation of ligand

Lipinski’s rules were used to predict 25 compounds Based on docking. Pub Chem compound database was used to derive the structures of the bioactive compound.

#### Target protein retrieval

The selection of aldose reductase (PDB ID: 2FZD) for diabetic activity and Cadherin-11 (PDB ID: 6CGB) for arthritis was based on their interaction with *L.edodoe* bioactive molecules. From the Protein Data Bank (PDB) and retrieved the 3D crystal structures of the proteins.

#### Target protein preparation

In this study, proteins were processed, prepared, and converted into protein models using Discovery Studio software. As a result of removing water molecules from the structures, protein structures were determined by X-ray crystallography. Moreover, Discovery Studio software was used to identify potential binding sites for potential compounds, which could lead to further advancements in drug discovery.

#### Docking.

The ligands and target proteins were docked using AutoDock Vina. Docking generated the ligand conformations, and the final refinement of the ligand pose was done. Using the boundary line of the grid box or entering values in the appropriate box, the size and coordinates of the grid box were adjusted. The coordinates and distances of the × , y and z axes of the grid boxes of each protein molecule were center x (36.74), center y (132.62) and center z (20.60). While the size of x size 84.53, y size 88.93 and z size 24.0 The docking procedure was then worked at the exhaustiveness of 8, and set to generate only the lowest energy pose. Bioactive compounds were docked into target proteins to calculate the best docking score^28^.

#### Molecular docking analysis.

Ligands and proteins are docked to find the highest effective orientation. A molecular docking analysis of *L. edodes* bioactive compounds was conducted. Using BIOVIA Discovery Studio, bioactive compounds were docked to calculate binding energies (version 2021) In AutoDock Vina, aldose reductase and sulfatase-2 are used as target proteins. After preparing the ligands and target proteins, AutoDock Vina automatically docks them together. Discovery Studio Visualizer analyzes binding energies, binding contacts, and docking data for each ligand^29^.

#### Deformability, B-factor, and covariance computation

Molecular dynamic simulation complexes have been analyzed for deformability, B-factor, covariance, and root-mean-square fluctuations to determine any residues that remain unstable or deformed after coarse-grained simulations. A deformability analysis, B-factor analysis, and covariance analysis were performed using IMODS software^30^.

#### ADMET study

Using ChemDraw Ultra 8.0, the structures of selected compounds were illustrated to analyze their pharmacokinetics (absorption, distribution, metabolism, and excretion). To predict the drug-like and pharmacokinetic properties of the selected compounds, the legends were converted into SMILES format and the ADME tool was used by an online server (http://www.swissadme.ch/, accessed on 15 December 2022)^31^.

#### QSAR-modelling

Using Cloud 3D-QSAR, an online tool that analyzes molecule-to-molecule interactions in biological systems, the model was developed in 3D. The half-maximal inhibitory value of each ligand or compound is manually calculated in nanometers^32^.

#### Statistical analysis

The results are revealed as Mean SD in the Figures. A two-way ANOVA was performed using Graph Pad Prism (San Diego, CA, USA) software. The graphs’ values are all shown as mean SE. p < 0.05 and p < 0.001 (***) when compared to the control, respectively.

### Ethical approval

All the methods were performed in accordance whit relevant institutional guidelines and regulations.

## Results

### FT-IR analysis

**Figure 1:**
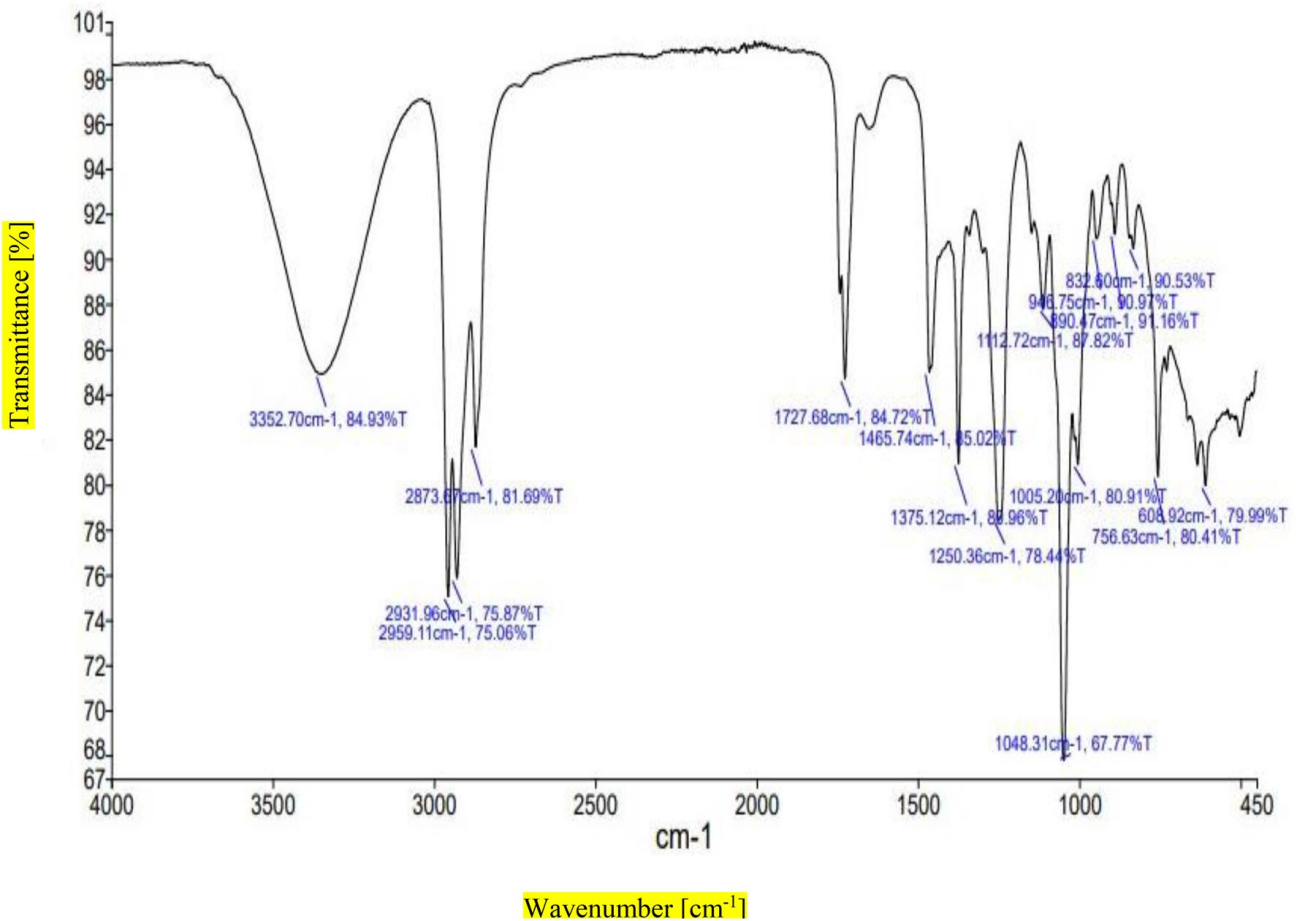
FT-IR spectrum of *n*-butanol extract.

The presence of functional groups was subsequently confirmed using FT-IR analysis. FT-IR spectrum in Fig. 1 showed transmittance and wavelength values, transmittance give the quantitative analysis and physical meaning to show the functional group. FT-IR spectra of *n*-butanol extract contain the presence of the N–H stretching group at 3352.70 cm^−1^ and carbonyl (C-H stretching) group at 2959.11 cm^−1^, 2931.96 cm^−1^ and 2873.67 cm^−1^ while at 1727.68 cm^−1^ (C = O stretching) shown in Fig. 1.

### GC–MS analysis

**Figure 2:**
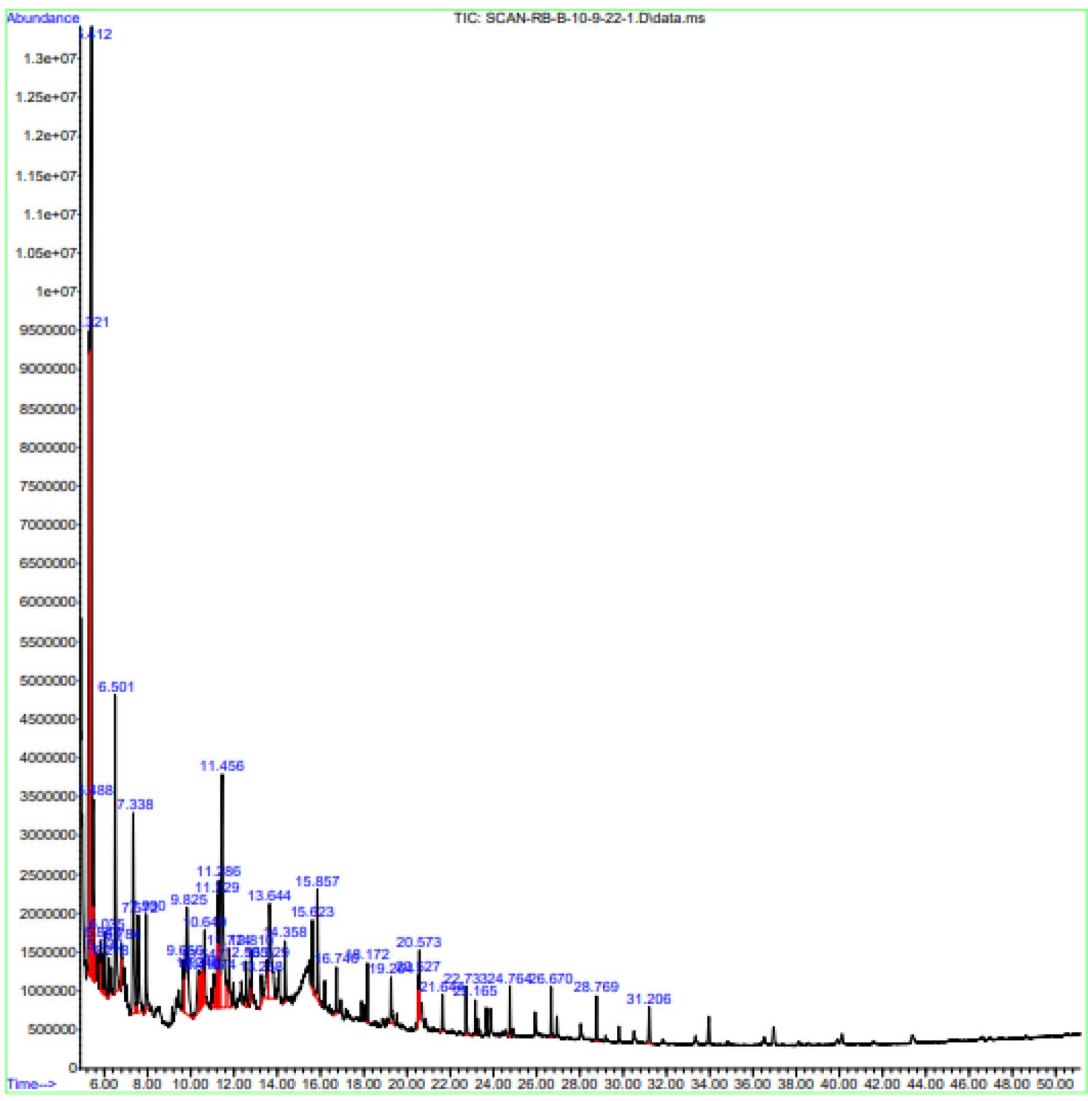
GC–MS chromatogram of *n*-butanol extract of *L. edodes*.

Different bioactive compounds were identified in *n*-butanol extract of *L. edodes* by GC–MS analysis. The prevailing compounds were 2-pyrrolidinone Silane,[(1,1-dimethyl-2 propenyl)oxy]dimethyl; Morphine, 2TMS derivative; Benzoic acid, methyl ester 1-(2-methoxy-1-methylethoxy)-2-propanol, TBDMS derivative, which were spotted in highest range in *n*-butanol crude extract of *L. edodes* (Fig. 2 and Table 1).

**Table 1:** Metabolites identified from *L. edodes n*-butanol extract by GCMS analysis*.*

Peak no	Retention time (min)	Compound names/hits names	Molecular formula	Molecular extract mass (M)	Peak area %
	5.320	2-Pyrrolidinone	C_4_H_7_NO	85.10	12.40
	5.411	2-Pyrrolidinone	C_4_H_7_NO	85.10	25.54
	5.486	Benzoic acid, methyl ester	C_8_H_8_O_2_ or	136.15	2.55
	5.844	*N*-acetyl-d-glucosamine	C_8_H_15_NO_6_	221.21	0.65
	5.930	2-Acetamido-2-deoxy-alpha-d-glucopyranose	C_7_H_15_NO	129.20	0.37
	6.037	Formic acid, (2-methylphenyl)methyl ester	C_7_H_12_O_2_S_2_	192.3	1.24
	6.219	[2-(2*H*-tetrazol-5-yl)cyclopropyl]methanamine	C_2_H_5_N_5_	99.10	0.46
	6.502	Silane,[(1,1-dimethyl-2 propenyl)oxy]dimethyl	C_7_H_15_OSi	143.28	5.12
	6.786	*N,N*-dimethyl formamide ethylene acetal	C_5_H_11_NO_2_	117.15	0.12
	7.337	1,4:3,6-dianhydro-.alpha.-d-glucopyranose	C_6_H_8_O_4_	144.12	4.75
	7.572	1-Allkyl(dimethyl)silyloxypropane	C_8_H_18_OSi	158.31	2.27
	7.930	1,1-Diisobutoxy-isobutane	C_12_H_26_O_2_	202.33	1.63
	9.658	*N,N’,N*’’Trimethyldiitrimethylenetriamine	C_9_H_23_N_3_	173.30	1.02
	9.824	5,6-Diamino-1,3-dimethyluracil	C_6_H_10_N_4_O_2_	170.17	4.62
	10.348	1,3-Dioxane-2-propanol, 2-methyl	C_8_H_16_O_3_	160.21	1.33
	10.417	1-Hexanamine	C_6_H_15_N or CH_3_(CH_2_)_5_NH_2_	101.19	0.77
	10.546	Formic acid, 1-methylpropyl ester	C_5_H_10_O_2_	102.13	1.29
	10.642	Eugenol	C_10_H_12_O_2_	164.20	1.11
	11.075	1,4-Butanediamine	C_4_H_12_N_2_	88.15	0.79
	11.230	Formamide, *N*-(cyanomethyl)-	C_3_H_4_N_2_O	84.08	1.57
	11.284	Diisopropyl(methoxy)silane	C_7_H_17_OSi	145.29	2.06
	11.455	*N*.omega.-nitro-l-arginine	C_6_H_13_N_5_O_4_	219.20	8.74
	11.776	Carbamic acid, ethylnitroso-, ethyl ester	C_5_H_10_N_2_O_3_	146.14	1.28
	12.562	Silane, ethenyldimethoxymethyl-	C_9_H_22_O_2_Si	190.35	0.75
	13.258	2-Amino-*N,N*-dimethylethanesulfonamide	C_4_H_12_N_2_O_2_S	152.22	0.37
	13.530	2-Butanamine, *N*-methyl-*N*-nitroso	C_5_H_12_N_2_O	116.16	1.30
	13.643	3-Buten-1-ol	C_4_H_8_O	72.11	3.90
	14.359	D-Glycero-D-galacto-heptose	C_7_H_14_O_7_	210.18	0.95
	15.622	Cyclooctasiloxane, hexadecamethyl	C_16_H_48_O_8_Si_8_	593.2	0.84
	15.857	l-Glutamic acid	C_5_H_9_NO_4_	147.13	1.77
	16.740	1-(2-methoxy-1-methylethoxy)-2-propanol, TBDMS( tert-butyl dimethyl silyl) derivative	C_13_H_30_O_3_Si	262.46	0.73
	18.173	Cyclononasiloxane, octadecamethyl	C_18_H_54_O_9_Si_9_	667.4	0.67
	19.264	Morphine, 2TMS(trimethylsilyl )derivative	C_23_H_35_NO_3_Si_2_	429.7	0.86
	20.527	Oxazepam, 2TMS(trimethylsilyl) derivative	C_21_H_27_ClN_2_O_2_Si_2_	431.1	0.54
	20.575	Pentadecanoic acid, 14-methyl-, methyl ester	C_17_H_34_O_2_	270.5	0.76

### LC–MS analysis

Butanol extract was passed through silica gel column to attain column fraction for partial purfication of bioactive compounds intital screening was carried out by performing Thin Layer Chromatography before subjected to LC-MS. The results of LC-MS analysis of the *n*-butanol extract of *L. edodes* illustrated the presence of total four different phytoconstituents at different retention times. Phytoconstituents were identified through LC-MS that might be responsible for pharmacological activities. The compounds were recognized based on retention time and molecular mass by comparing with Mass Bank Data Base and HMDB.

**Figure 3:**
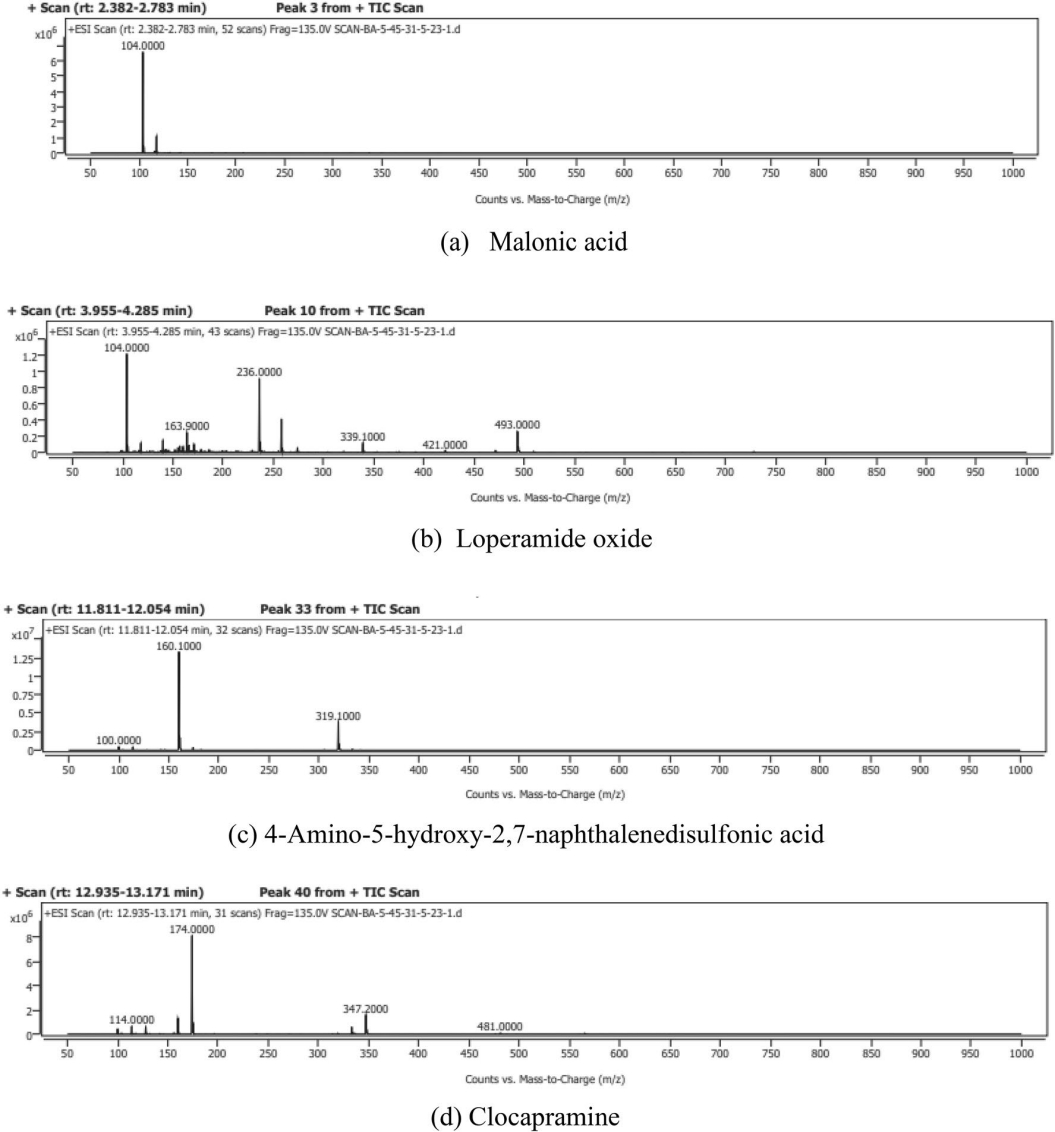
LC–MS anlysis of *n*-butanol extract of *L. edodes*.(**a**)Malonic acid (**b**) Loperamide oxide (**c**) 4-Amino-5-hydroxy-2,7-naphthalenedisulfonic acid (**d**) Clocapramine.

The LC-MS chromatogram detected compounds of *n*-butanol extract of *L. edodes* of the detected compounds shown in (Figure 3). It was observed that the different peaks were obtained at different retention times. In this, the highest peak is at the retention time of 13.077, 11.858 followed by 4.120, 2.477 belonging to the compound acid Clocapramine, 4-Amino-5-hydroxy-2,7-naphthalenedisulfonic, Loperamide oxide and Malonic acid (Table 2).

**Table 2:** LC–MS analysis of n-butanol extract of L. edodes.

Sr.no	Compound	Chemical formula	*M/z*	Monoisotopic mass	Peak num	Retention time	Production	Area%
1	Malonic acid	C_3_H_4_O_4_	104.1	104	3	2.477		75.01
2	Loperamide oxide	C_29_H_33_ClN_2_O_3_	493	492	10	4.120	421.0,339, 236,163	23.89
3	4-Amino-5-hydroxy-2,7-naphthalenedisulfonic acid	C_10_H_9_NO_7_S_2_	319.3	319	33	11.858	160,100	97.31
4	Clocapramine	C_28_H_37_ClN_4_O	481	480	40	13.077	347.2,174,114	67.76

Malonic acid is a dicarboxylic acid with structure. Malonates are ionized forms of malonic acid, as well as its esters and salts. The compound malonic acid is used to prepare cinnamic acid, which is used to make cin metacin, an anti-inflammatory compound.

This compound belongs to the class of organic compounds known as diphenylmethanes with peak number 10. It is used as an anti-inflammatory.

4-Amino-5-hydroxy-2,7-naphthalenedisulfonic acid 2-naphthalenesulfonic acid substituted with an amino group at position 7 and a hydroxy group at position 4 is known as an aminonaphthalenesulfonic acid. It has a role as a metabolite. As a member of the naphthol family, it is an aminonaphthalenesulfonic acid. Clomipramine is a tricyclic compound detected in *n*-butanol subfraction with peak number 40 with 481 molecular weight with product ions of 37,174 and 114.

### Biological activities

#### Anti-inflammatory

**Figure 4:**
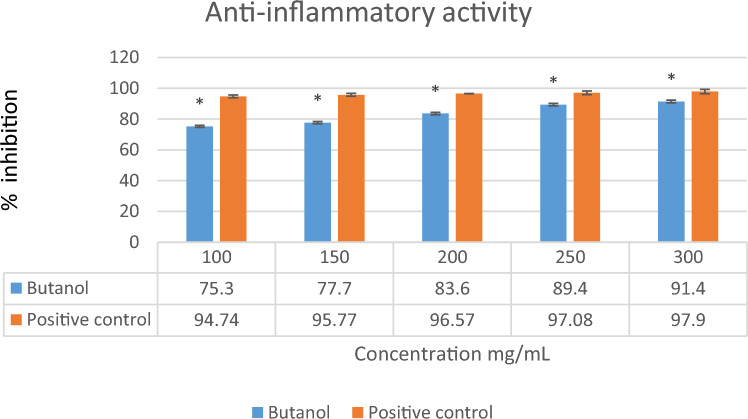
Percentage inhibition of protein denaturation of *n*-butanol extract of *L. edodes* Vertical bar represents Mean ± SD. The values are expressed in the Mean ± SD of triplicates and all values were statistically significant p < 0.05 (*).

The present study demonstrated the anti-inflammatory potential of *n*-butanol extract of *L. edodes* on denaturation of protein inhibition in vitro, as shown in Fig. 4. As a result of 300 mg/mL concentration, 91.4% inhibition was measured, which is similar to 97.9% inhibition measured with the positive control (Diclofenac sodium). In the *n*-butanol extract, 75.3% inhibition was observed at a concentration of 100 mg/mL shown in Fig. 4.

#### Antioxidant activity

**Figure 5:**
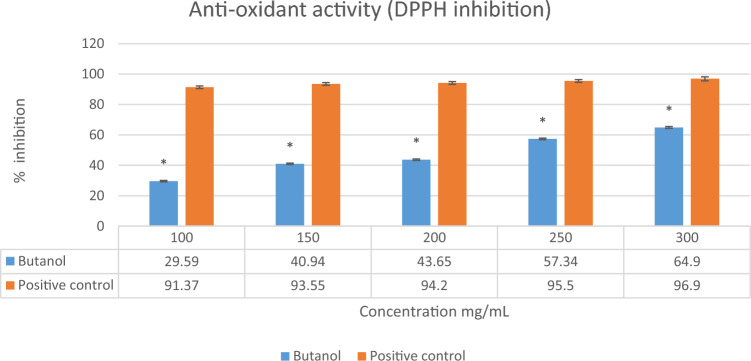
Free DPPH radical scavenging activity of *n*-butanol extract of *L. edodes*. The vertical bar represents Mean ± SD. The values are expressed in the mean ± SD of triplicates and all values were statistically significant p < 0.05 (*).

The *n*-butanol extract of *L. edodes* was analyzed for antioxidant potential by DPPH. Results showed that the scavenging activity of *n*-butanol extract on antioxidant assay was concentration dependent i.e., increases with increasing concentration (100–300 mg/mL). The maximum concentration was 64.6% as compared to the positive control (ascorbic acid) which was shown in Fig. 5.

#### Anti-diabetic activity

**Figure 6:**
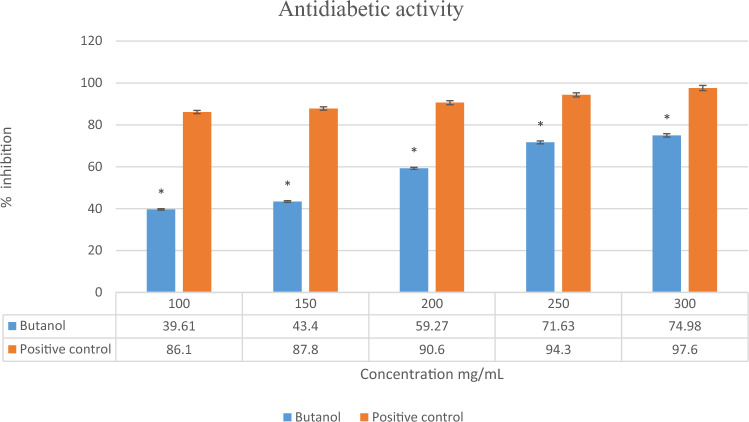
Antidiabetic activity of *L. edodes* *n*-butanol extract. The vertical bar represents Mean ± SD. The values are expressed in the Mean ± SD of triplicates and all values were statistically significant p < 0.05 (*).

This study analyzed the *n*-butanol extract for α-amylase enzyme inhibition by DNSA and starch iodine assay*.* The concentration of *L. edodes n*-butanol depended on the inhibition of α-amylase shown in Fig. 6. The butanol crude extract showed maximum inhibition at 300 mg/mL (74.98%), indicating anti-diabetic potential. As the concentration decreases, the anti-diabetic activity also decreases and is minimal at the lowest concentration. The positive control metformin showed a maximum (97.6%) at 300 mg/mL concentration, as shown in Fig. 6.

#### In-silico anti-diabetic activities with Aldose Reductase

**Figure 7:**
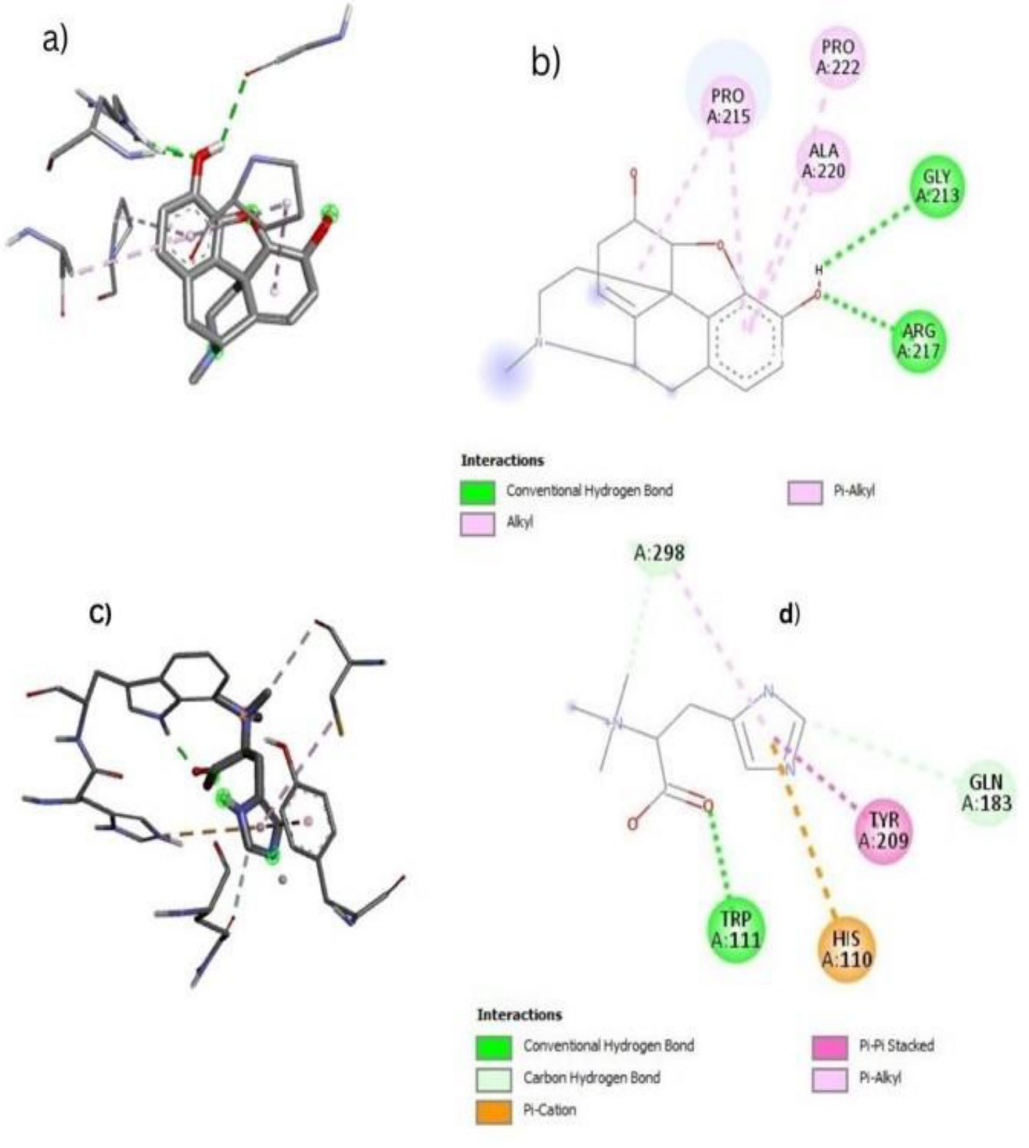
Molecular docking of aldose reductase (PDB ID: 2FZD) diabetic proteins with bioactive compounds (**a**) show the 2D image of morphine (**b**) show the 3D image of morphine (**c**) 2D image of N,N,N-trimethyl-histidine (**d**) 3D image of N,N,N-trimethyl-histidine.

Molecular docking of compounds with aldose reductase (PDB ID: 2FZD) was performed, and results show that the morphine and *N,N,N*-trimethyl-histidine have the highest binding affinity 7.0 and − 5.7, respectively (Fig. 7) and highest binding scores compounds were given in Table 3.

**Table 3:** Molecular docking of bioactive compounds against aldose reductase.

Sr. No	Compounds	Docking score (kJ/mol)
1	Morphine	**− **7.0
2	*N,N,N*-trimethyl-histidine	− 5.7
3	5-Cyclopropyl-4-pentene-1-ol	− 5.1
4	Alpha d-Galactose	− 5.0
5	1,3-Dioxolane-2-propanol, 2-methyl-	− 4.9
6	Methyl benzoate	− 4.9
7	5,6-Diamino-1,3-dimethyluracil	− 4.9
8	D-Glycero-D-galacto-heptose	− 4.8
9	2-Nitrophenethyl alcohol, TMS derivative	− 4.6
10	2-Nitrophenethyl alcohol, TMS derivative	− 4.6

### In-silico drug designing against arthritis

#### Molecular docking

**Figure 8:**
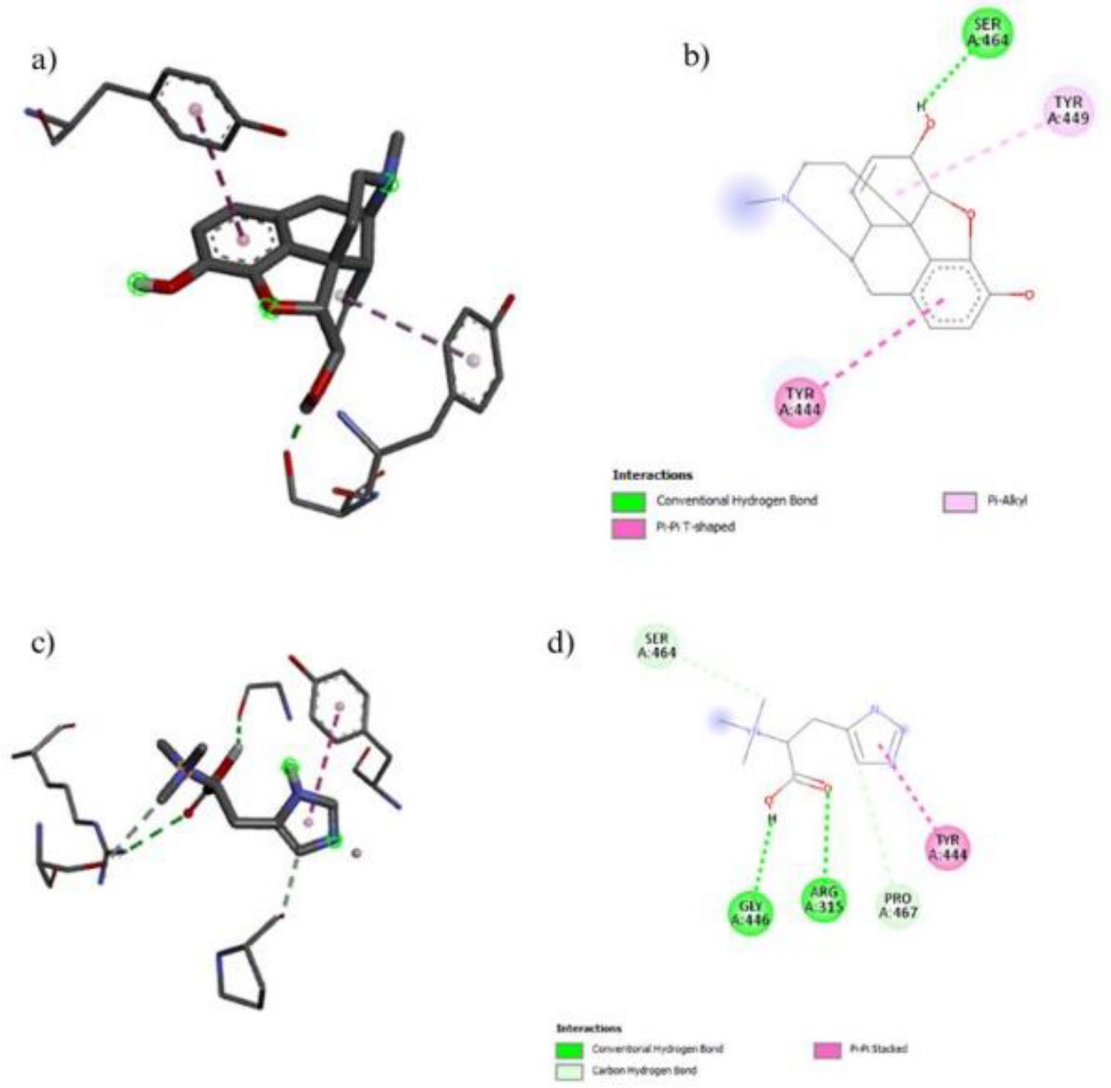
Molecular docking of sulfatase‑2 proteins of arthritis with bioactive compounds (**a**) shows the 2D image of morphine (**b**) shows the 3D image of morphine (**c**) 2D image of N,N,N-trimethyl-histidine (**d**) 3D image of N,N,N-trimethyl-histidine.

**Figure 9:**
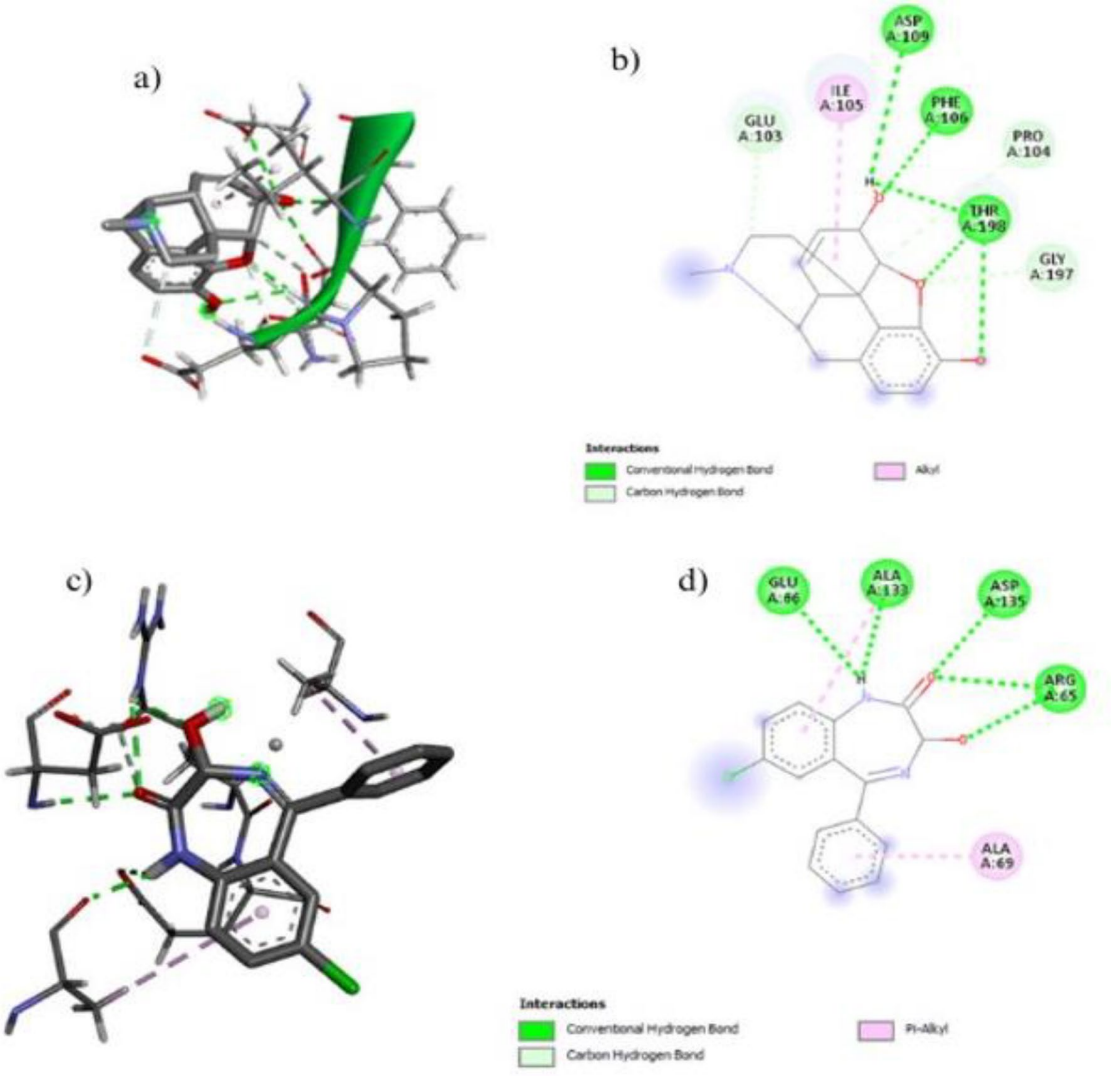
Molecular docking of cadherin -11 protein of arthritis with bioactive compounds (**a**) shows the 3D image of morphine (**b**) shows the 2D image of morphine (**c**) 3D image of N,N,N-Trimethyl-Histidine (**d**) 2D image of N,N,N-Trimethyl-Histidine.

The molecular docking of five compounds was performed with targeted arthritis protein cadherin-11 and sulfatase‑2 docking scores Fig. 8 and Fig. 9. Among all compounds, Morphine and *N,N, N*-trimethyl-histidine show high docking energy − 7.9 and − 7.5 against sulfatase‑2 respectively Morphine and Oxazepam show high docking energy − 8.0 and − 6.5 shown in Fig. 8 and Table. 4.

**Table 4:** Molecular docking of bioactive compounds against sulfatase‑2.

Sr.No	Compounds	Sulfatase‑2 kJ/mol	Cadherin-11 kJ/mol
	Morphine	− 7.9	− 8.2
	Oxazepam	− 7.4	− 6.5
	*N,N,N*-trimethyl-histidine	− 7.5	− 5.8
	D-Glycero-D-galacto-heptose	− 5.7	− 5.1
	1-*N*-acetyl-beta-D-glucosamine	− 5.6	− 5.1
	Eugenol	− 5.6	− 5.4
	Methylbenzoate	− 5.5	− 5.1
	Formic acid, (2-methylphenyl)methyl ester	− 5.4	− 5.2
	5,6-Diamino-1,3-dimethyluracil	− 5.3	− 5.9
	D-Glycero-D-galacto-heptose	− 5.1	− 4.9

### IMODS simulations

#### Molecular dynamic simulation

**Figure 10:**
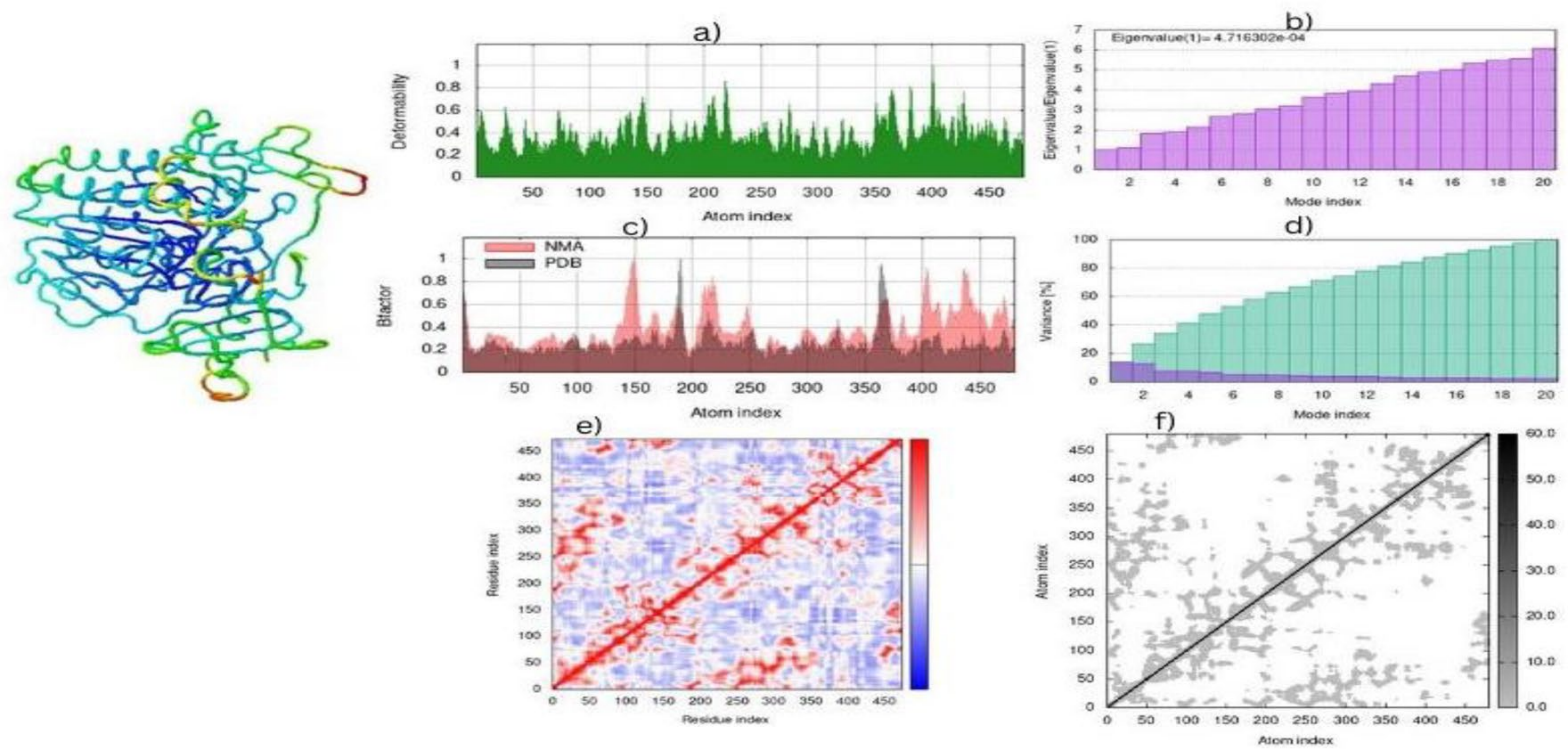
In this Molecular Dynamics simulation, morphine docked with sulfatase-2 (**a**) depicts the compound docked (**b**) indicates the structure has low deformability (**c**) shows the B-factor, (**d**) Eigenvalues are shown, and (**e**) shows the variance explained in green and red (**f**) and (**g**) show the covariance and elastic network of the complex.

By using the force field of the complex concerning a time interval, iMods simulates the molecular dynamics of the complex. At each level of residue capacity, the complex model displays less deformation. The Eigenvalue of Cadherin-11 complex is 2.80269e-05. As illustrated in the Figure, residues in a highly related region with low RMSD exhibit a better interaction as a result of the molecular dynamic simulation shown in Fig. 10.

#### SwissADME

**Figure 11:**
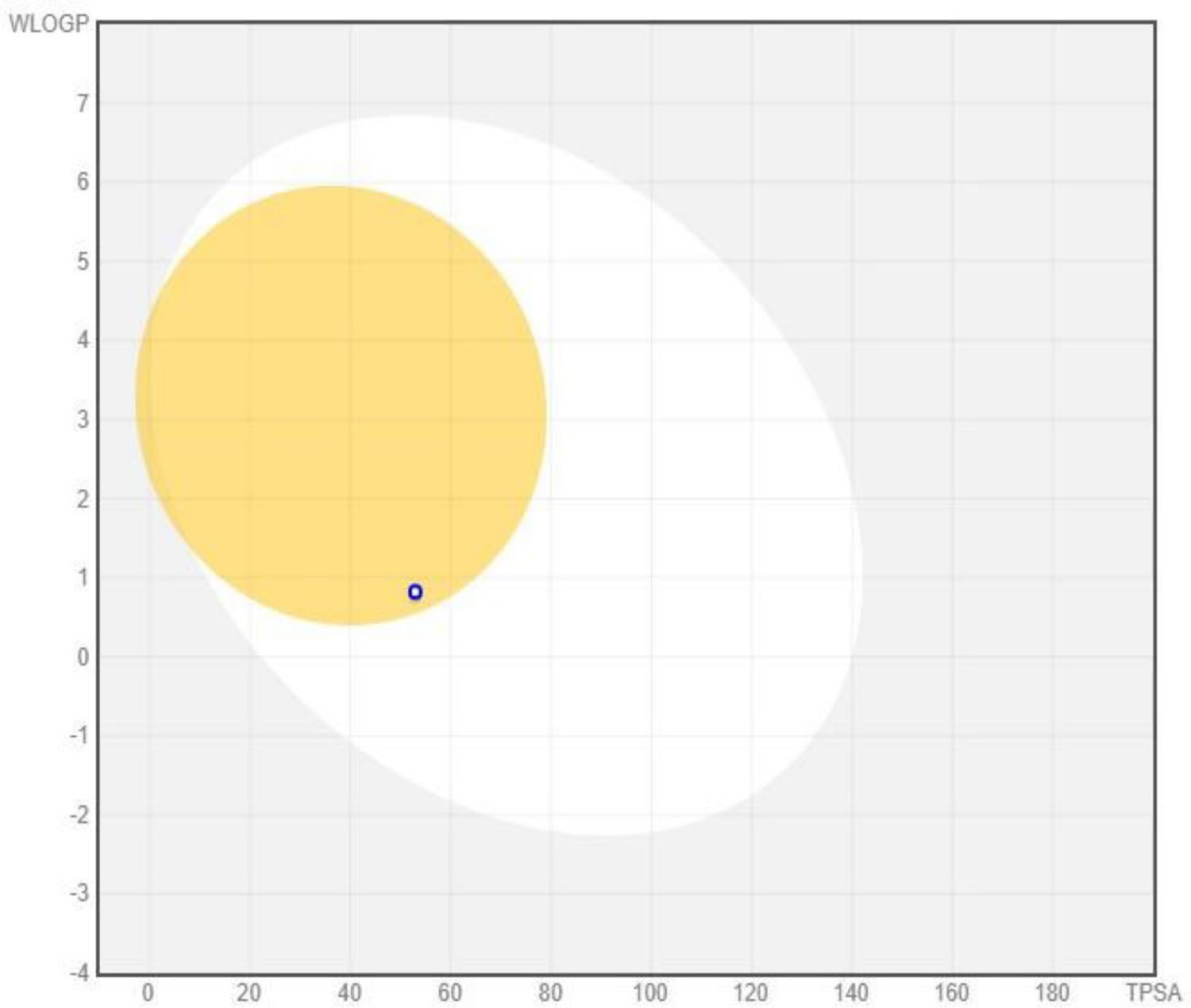
A morphine compound was analyzed in a boiled showed the dependence of lipophilicity on the polarity of the studied molecules.

After docking results, SwissADME characterized the compounds with the highest inhibitory affinity against Cadherin-11. It is used to determine the toxicity and properties of a compound. It is possible to calculate logS and logP as well as different other drug-likeness rules using Swiss ADME’s multiple calculation approaches. In comparison to all compounds, morphine achieved the highest docking score and ADMET properties of − 2.30, with excellent water solubility and low GI absorption. The drug analysis shows that Lipinski and Veber’s rule applies. The analysis of BOILED eggs is shown in Fig. 11.

#### QSAR Modeling

**Figure 12:**
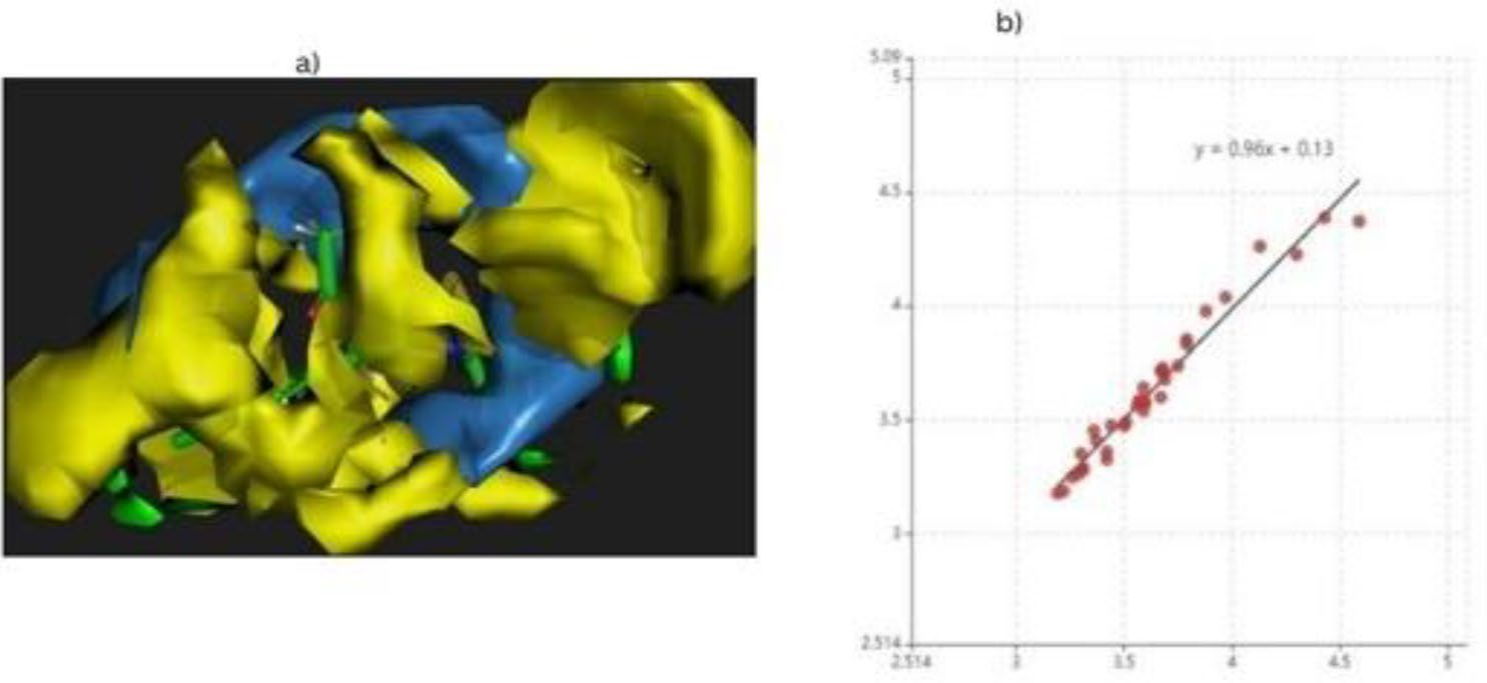
The QSAR cloud shows the following results (**a**) a Contour map of the compound (morphine) (**b**) Graphical representation of data of bioactive compounds.

Using Cloud 3D-QSAR, we modeled 3D QSAR in biological systems and analyzed the relationships between molecules and their behavior. Each ligand or compound in the dataset had an IC50 value determined manually in nm. All compounds give a higher r20.9993 and a smaller q2 − 0.80663 than model number 7 morphine. A steric hindrance analysis and electrostatic interaction force were conducted to obtain the results. Moreover, it determines the pKi value of compounds, and experimental data indicates that morphine is the most effective ligand. The graphical representation of data and the contour map structure of the best drug candidate are shown in Fig. 12.

## Discussion

Mushrooms have been used as medicines by humans for at least 5,000 years^33^ globally, the prevalence of diseases is increasing due to two main factors: inadequate nutrition associated with modern lifestyles and an increase in average life expectancy^34^. Secondary metabolites derived from medicinal mushrooms have a key role in improving health status from ancient times in Asian medicine. Numerous mushroom species have been used to treat immune-based diseases, such as Multiple Sclerosis (MS)^35^. There are several secondary metabolites present in mushrooms that can be used therapeutically, such as lactones, terpenoids, alkaloids, antibiotics, and metal-chelating agents^36,37^. FT-IR spectrum of *n*-butanol extract shows different functional groups including N–H stretching group, Alkane (C-H stretching) group, (C = O stretching) shown in Fig. 1. 0.GCMS analysis showed 35 biometabolites among these Morphine, oxazepan and *N,N,N*-trimethyl-histidine is highly significant . The present study also indicates the presence of 2-Pyrrolidinone (12.40%). Silane, [(1,1-dimethyl-2 propenyl) oxy] dimethyl (5.12%); Morphine, 2TMS derivative (0.86%); Benzoic acid, methyl ester 1-(2-methoxy-1-methylethoxy)-2-propanol (2.55%), present in the fruiting bodies of mushroom *L. edodes* that demonstrated in Fig. 2 and Table 1. Previous cited literature showed *L. edodes* have potential biometabolites which might be responsible for biological potential. Previous studies correlate with current study which explained 297 different metabolites of two strains of *L. edodes* with different tolerances at different durations of high temperature including amino acids, sugars and glycols like Proline, Gallic acid, 3-Hydroxypropionic acid, Dibenzofuran, Aminomalonic acid, Oxalic acid which indeed is good agreement with present study^38^. There was a higher percentage composition associated with the different biological activities among the bioactive components. Although inflammation serves a protective role, many diseases, such as atherosclerosis, arthritis, cancer, and ischemic heart disease, have etiological causes in inflammatory processes^1^. The purpose of this study was to investigate the anti-inflammatory properties of *n*-butanol mushroom extracts and their bioactive metabolites. According to the results, *n*-butanol has excellent anti-inflammatory activity at 91.4% as at 300 mg/mL, GC–MS-based identified metabolites showed an impressive maximum inhibitory concentration (91.4%) in comparison with the positive control Diclofenac sodium that showed a higher percent stabilization, at 97.9%. Previous cited literature also showed that mushroom species have significant anti-inflammatory potential^39^. Living conditions and organisms can be reflected in the metabolism. As metabolite detection and identification methods continue to advance, interest in metabolic control in response to abiotic stress has grown significantly. Numerous studies have focused on the role of metabonomics in the defence mechanism in challenging conditions, mostly using some model plants, crop varieties, and yeasts in fungus^40,41^. In previous study, sophisticated GC–MS metabonomics conducted both domestically and internationally yielded metabolites. Using a Venn diagram to intersect differential metabolites that were significantly altered in each treatment, 74 metabolites with notable variations in 18 species of mushrooms were found, but the mutant strain 18N44 included 106 significant differential metabolites^42^. Following a thorough analysis of the two strains, 47 important differential metabolites that underwent significant alteration were identified. By using KEGG mapping, several maps of metabolic pathways were produced. Pathway enrichment analysis identified five different metabolic pathways. Amino acids, sugars or glycols, intermediate products of glycolysis, and the TCA cycle were the primary enriched compounds. In this study, 35 metabolites were identified in *L. edodes* mushrooms, they play an important role in the metabolism of living organisms such as amino acid, sugar or glycols, intermediated products of glycolysis and TCA cycle of living organism^42^.

Butanolic extract was further fractionated to purify biological active compounds by loading silica gel 400–600 µm pore size in a glass column by selecting different solvents to set polarities according to the nature of the fraction to collect the column fraction for further purification of secondary metabolites by passing extract through the column. Partially purified biometabolites were determined in the collected fraction by performing LC–MS.LC–MS investigation was accomplished for determination of high molecular weight and nonvolatile bioactive compounds in the *n*-butanol extract passed through column chromatography. Present results depicted different peaks at different retention times showed valuable biometabolites including Clocapramine, 4-Amino-5-hydroxy-2,7-naphthalenedisulfonic, Loperamide oxide and Malonic acid illustrated in above Table 2. Previous literature showed significant amount of bioactive compounds in different mushrooms species^43^.Above said study correlate with previous literature which showed LC–MS analysis of brown film of *L. edodes* mycelia which depicted total of 236 nonrepetitive different metabolites, in which 61 were common differential metabolites. Many differential metabolites were first identified by means of an untargeted LC–MS-based metabolomics approach in the brown filem of *L. edodes*, which also shows the variety and richness of the metabolites in brown film of *L. edodes*^44^. Previous cited literature illustrated these bioactive compounds possess significant pharmacological activities 4-Amino-5-hydroxy-2, 7-naphthalenedisulfonic acid possess antioxidant, anti-inflammatory and anticancerous activity while Malonic acid as anti-inflammatory and antioxidant effect^45^.

As a comparison to the previous literature^15^, our results impacted good anti-inflammatory activity. Most diseases in humans are caused by the accumulation of free radicals. Antioxidants are responsible for scavenging free radicals and minimizing their destructive effects^16^. In the human body, reactive oxygen species are constantly formed by metabolism and diseases, resulting in the damage of tissues and causing degenerative diseases and extensive lysis of tissues. To overcome oxidative damage, there are several synthetic drugs available that can be used, but these drugs are associated with many adverse side effects. Alternative solutions to such side effects include consuming natural antioxidants in the form of food supplements and traditional medicine to avoid these side effects^46^. The current study was appraised to investigate the antioxidant potential of *n*-butanol extracts of shiitake mushroom (*L. edodes*) to protect the body from free radicals. The *n*-butanol extract of *L. edodes* at 300 mg/mL was shown in Fig. 5 to have a significant inhibitory potential (64%),that indicates the potent antioxidant behavior of the secondary metabolites. Previous cited literature correlated with current study showed significant antioxidant potential in *L. edodes* prepared extract which is 45–90% of ethanolic extract^39^. The presence of β-glucans and phenolic compounds in *Lentinus edode*s suggests this mushroom could be a suitable candidate for nutritional supplementation^47^. The medicinal properties of edible mushrooms include antitumor activity related to their β-D-glucan content; and antidiabetic from decreasing blood glucose due to the presence of bioactive substances (including phenolics and ergothioneine)^48^.

The biological assay was conducted with *n*-butanol extract, which showed 74% anti-diabetic activity at 300 mg/mL. This study was performed to evaluate the antidiabetic effects of extracts in vitro and in silico analysis. Morphine shows the best anti-diabetic compound against Aldose reductase and thus was proved from molecular docking. The reported study shows that the analgesic effect of morphine is reduced in hyperglycemia, based on experimental and clinical observations which is 60–82%^49^. An inflammatory disorder with a high mortality rate, rheumatoid arthritis is considered a systemic inflammatory disorder. The prevalence of RA has increased the economic burden, despite the persistent reduction in RA mortality. It’s an autoimmune chronic degenerative disease that affects joints and extra-articular tissue^50^ Extracellular sulfatase-2 (Sulf-2) influences receptor–ligand binding and subsequent signaling by chemokines and growth factors, although its role in inflammatory cytokine signaling in Rheumatoid arthritis (RA) has not been explored^51^.

Drug discovery in the modern era is most commonly achieved through computer-aided drug design (CADD)^52^. Molecular docking is a method of predicting the experimental binding affinity of inhibitors (ligands) to proteins. Drug-likeness reveals the potential suitability of a ligand for a biological system, whereas molecular docking reveals the interaction of ligands with active sites of protein^53^. Docking was done against sulfatase-2 and cadherin-11 the high score showed good interaction between morphine and the receptor that demonstrated in Table 4.The current docking results are similar with previous study reported by^54^ . Those results show morphine is effective against arthritis.

Drug development and agrochemical design have benefited from quantitative structure–activity relationships (QSAR)^55^.QSAR can be used to design drugs using genetic algorithms and evolutionary algorithms. Research has shown that the many applications of QSAR are identification and conformation searches of QSARs, receptor docking, variable selection, and pharmacophore and receptor elucidation. The R2 QSAR value (0.9993) and Q2 (− 0.8066) analysis of the good value ligand. Normal mode analysis mobility reveals the large-scale B-factor and mobility as well as the stability of the molecules. Further molecular simulation (MD) exposed the internal coordinate’s analysis depending on the protein–ligand structural interactions^56^. IMODS simulations were performed for the top first compounds of cadherine-11 to assess the stability of the docked complexes between phytochemicals and proteins through strong hydrogen bonds, as shown in Fig. 9. Based on ADME and drug-like properties, the molecules are moderately bioavailable through the gastrointestinal tract, but not permeable through the brain. In the bioavailability radar, six physicochemical properties of a drug are considered: saturation, Fig. (10) BOILED-egg image of morphine complex. In the yellow region, brain penetration was high, the white region was high, and the red dot showed that the compound wasn’t a substrate of the P-gps because of their polarity, flexibility, size, lipophilicity, and solubility. It appears that morphine may be beneficial for ruminated arthritis under In silico analysis. The ADME and drug-like properties of morphine is highly potential against different ailments which was previously reported studies^57–60^. Clinical breakthroughs in the treatment and prevention of infectious diseases are being achieved through the targeting of bacterial diseases. Further research into these potential targets is necessary in order to identify novel approaches to the development of future drugs and treatments.

## Conclusion

The findings of this study revealed the possible use of *L. edodes* butanolic extract in food and nutraceutical applications such as anti-inflammatory, antioxidant and anti-diabetic which might be possible due to presence of active biometabolites. FT-IR, GC–MS and LC–MS were used to detect these bioactive compounds and their therapeutic potential was further assessed by molecular docking principles following an insilico approach for *n*-butanol extracts. In which 15 compounds were docked out of which 10 gives best possible binding energies. In silico virtual screening showed that morphine is effective against ruminated arthritis. The results show that the extract contains a wide range of bioactive compounds, which may be beneficial for various therapeutic applications Therefore mushrooms butanolic extracts is an ideal candidate for developing new drugs to combat emerging pathogenic bacteria, to tackle the global healthcare crisis.

## Data Availability

All the data has been included in the manuscript.
